# The Impact of Corticosteroids on Secondary Infection and Mortality in Critically Ill COVID-19 Patients

**DOI:** 10.1177/08850666211032175

**Published:** 2021-07-12

**Authors:** Lindsay A. Ritter, Noel Britton, Emily L. Heil, William A. Teeter, Sarah B. Murthi, Jonathan H. Chow, Emily Ricotta, Daniel S. Chertow, Alison Grazioli, Andrea R. Levine

**Affiliations:** 1 Division of Pulmonary and Critical Care Medicine, Department of Medicine, University of Maryland School of Medicine, Baltimore, MD, USA; 2 Division of Pulmonary and Critical Care Medicine, Department of Medicine, Johns Hopkins University School of Medicine, Baltimore, MD, USA; 3 Department of Pharmacy Practice and Science, University of Maryland School of Pharmacy, Baltimore, MD, USA; 4 Department of Emergency Medicine, Program in Trauma/Surgical Critical Care, R Adams Cowley Shock Trauma Center, University of Maryland School of Medicine, Baltimore, MD, USA; 5 Department of Surgery, Program in Trauma/Surgical Critical Care, R Adams Cowley Shock Trauma Center, University of Maryland School of Medicine, Baltimore, MD, USA; 6 Department of Anesthesiology and Critical Care Medicine, George Washington University School of Medicine, Washington DC, USA; 7 National Institute of Allergy and Infectious Diseases Division of Intramural Research, Epidemiology Unit, National Institutes of Health, Bethesda, MD, USA; 8 Critical Care Medicine Department, National Institutes of Health Clinical Center and Laboratory of Immunoregulation, National Institute of Allergy and Infectious Diseases, Bethesda, MD, USA; 9 National Institute of Diabetes and Digestive and Kidney Diseases, National Institutes of Health, Bethesda, MD, USA; 10 Department of Medicine, University of Maryland School of Medicine, Baltimore, MD, USA

**Keywords:** COVID-19, SARS-CoV-2, ICU, secondary infection, steroids, mortality

## Abstract

**Background:**

Corticosteroids are part of the treatment guidelines for COVID-19 and have been shown to improve mortality. However, the impact corticosteroids have on the development of secondary infection in COVID-19 is unknown. We sought to define the rate of secondary infection in critically ill patients with COVID-19 and determine the effect of corticosteroid use on mortality in critically ill patients with COVID-19.

**Study Design and Methods:**

One hundred and thirty-five critically ill patients with COVID-19 admitted to the Intensive Care Unit (ICU) at the University of Maryland Medical Center were included in this single-center retrospective analysis. Demographics, symptoms, culture data, use of COVID-19 directed therapies, and outcomes were abstracted from the medical record. The primary outcomes were secondary infection and mortality. Proportional hazards models were used to determine the time to secondary infection and the time to death.

**Results:**

The proportion of patients with secondary infection was 63%. The likelihood of developing secondary infection was not significantly impacted by the administration of corticosteroids (HR 1.45, CI 0.75-2.82, *P* = 0.28). This remained consistent in sub-analysis looking at bloodstream, respiratory, and urine infections. Secondary infection had no significant impact on the likelihood of 28-day mortality (HR 0.66, CI 0.33-1.35, *P* = 0.256). Corticosteroid administration significantly reduced the likelihood of 28-day mortality (HR 0.27, CI 0.10-0.72, *P* = 0.01).

**Conclusion:**

Corticosteroids are an important and lifesaving pharmacotherapeutic option in critically ill patients with COVID-19, which have no impact on the likelihood of developing secondary infections.

## Introduction

In March 2020, the United States saw a rapid surge in patients presenting with novel coronavirus-19 disease (COVID-19) due to the severe acute respiratory syndrome coronavirus 2 (SARS-CoV-2) virus. Clinically, these patients presented with fever, dyspnea, cough, sputum production, and hypoxemia. Twenty to 67% of patients hospitalized with COVID-19 will go on to develop acute respiratory distress syndrome (ARDS).^1,2^ Severe ARDS secondary to viral respiratory illnesses is well known and frequently complicated by secondary infection, or the occurrence of a second bacterial, fungal, or viral infection during or after the initial viral infection. In hospitalized patients with influenza, the incidence of secondary infection can exceed 30% and has been associated with an increased risk of death.^3^ The proportion of patients with secondary infection reported in COVID-19 admitted to the hospital has been variable and even reported to be low in some cohorts, with estimates ranging between 7%-15%.^4,5^ The exact proportion of secondary infection in critically ill patients remains inadequately defined but has been estimated to be between 5%-40%.^5–7^ Recent work in a cohort of critically ill and mechanically ventilated patients with COVID-19 demonstrated that 50.5% develop a second lower respiratory tract infection.^8^ Critically ill patients are at a higher risk of secondary infection due to their need for mechanical ventilation, central venous catheters, and prolonged hospitalizations, which place these patients at risk for developing hospital-acquired infections.^5,9–12^

The mortality benefit of corticosteroids remains heavily debated in ARDS in general but is increasingly demonstrated in COVID-19 related ARDS.^13–16^ Yet, clinicians often fear the immunosuppressive nature of corticosteroids leaving patients further prone to secondary infection.^17^ The Randomized Evaluation of COVID-19 Therapy (RECOVERY) trial demonstrated that dexamethasone reduced mortality in patients with COVID-19, particularly in mechanically ventilated patients, but performed no analysis of the impact of corticosteroids on the development of secondary infection.^13^ DEXA-COVID-19 (NCT04325061), CoDEX (NCT04327401), COVID STEROID (NCT04348305), and Steroids-SARI (NCT 04244591) intended to address secondary infection as an adverse event or secondary outcome but have closed or ceased enrollment after the publication of the RECOVERY trial leaving many of the findings unpublished or underpowered.^6,7,18,19^ When used in other viral pneumonias, corticosteroids have been shown to delay viral clearance, decrease immune system function, and contribute to secondary infections .^11,12,20–23^ Thus, the impact that corticosteroids have on the development of secondary infection in critically ill patients with COVID-19 remains unknown.

We hypothesize that secondary infection in critically ill COVID-19 patients is a frequent occurrence. We sought to describe the proportion of secondary infections in critically ill patients with COVID-19 and to determine whether corticosteroid use is associated with an increased rate of secondary infection. Furthermore, we sought to determine whether the development of a secondary infection is associated with mortality in critically ill patients with COVID-19.

## Materials and Methods

### Patient Selection

This was a retrospective cohort study of COVID-19 patients admitted to an Intensive Care Unit (ICU) at the University of Maryland Medical Center (UMMC) between March and June of 2020. The study was reviewed and approved by the University of Maryland, Baltimore, institutional review board (IRB). The requirement for written informed consent was waived by the IRB.

All patients had a confirmed diagnosis of SARS-CoV-2 by PCR testing. Patients were included in the study from the time that they arrived at their presenting hospital through death or hospital discharge. Patient demographics, laboratory, microbiology data, the use of COVID-19 directed therapies, and outcomes were abstracted by manual chart review from the medical record.

### Study Endpoints

The outcomes of the study were secondary infection and 28-day mortality. Secondary infection included bloodstream, respiratory, or urinary infection. Bloodstream infection was defined as positive blood culture without clear evidence of contamination. Respiratory infection was defined as a pathogen not considered to be a member of the normal respiratory flora isolated from the lower respiratory tract (sputum, tracheal aspirate, bronchoalveolar lavage). Urinary tract infection was defined as a pathogen obtained from the urine with greater than 100,000 colony forming units. Yeast in the sputum or urine was excluded. Cases of culture positivity were reviewed to confirm secondary clinical infection as determined by the expertise of the Infectious Disease consultant or Critical Care physician caring for the patient. Patients were determined to have secondary infection only when they had both culture positivity and sufficient clinical concern to warrant pharmacotherapeutic treatment. If individual patients developed multiple positive cultures during the hospital course, the first occurrence of a secondary infection was counted in the analysis. Patients who were co-infection on admission and/or died within 48 hours of admission were not included in the time to event analysis of mortality. Patients who had secondary infection prior to or within 2 days of receiving corticosteroids were excluded from the time to secondary infection analysis.

### Statistical Analysis

We calculated descriptive statistics of demographic and clinical characteristics and performed comparisons between groups using the Chi-square test of independence for categorical variables and the Mann Whitney U test for discrete variables.^24,25^ Time-to-event analyses were performed using univariate and multivariate proportional hazards models.

To assess the association between corticosteroid usage and secondary infection, we utilized Fine-Gray proportional hazards models (treating death as a competing risk).^26^ We adjusted for potential clinical confounders that were biologically relevant (age). When assessing for the association between secondary infection and mortality and steroids and mortality, we used a Cox proportional hazard model adjusted for age as a biologically relevant potential clinical confounder.^27^ Time to event analyses are depicted using Kaplan-Meier curves. Two-sided *P*-values of less than 0.05 were considered to indicate statistical significance. All analyses were performed using the R programming environment (v.4.0.2).

## Results

### Patients

**Figure 1.**
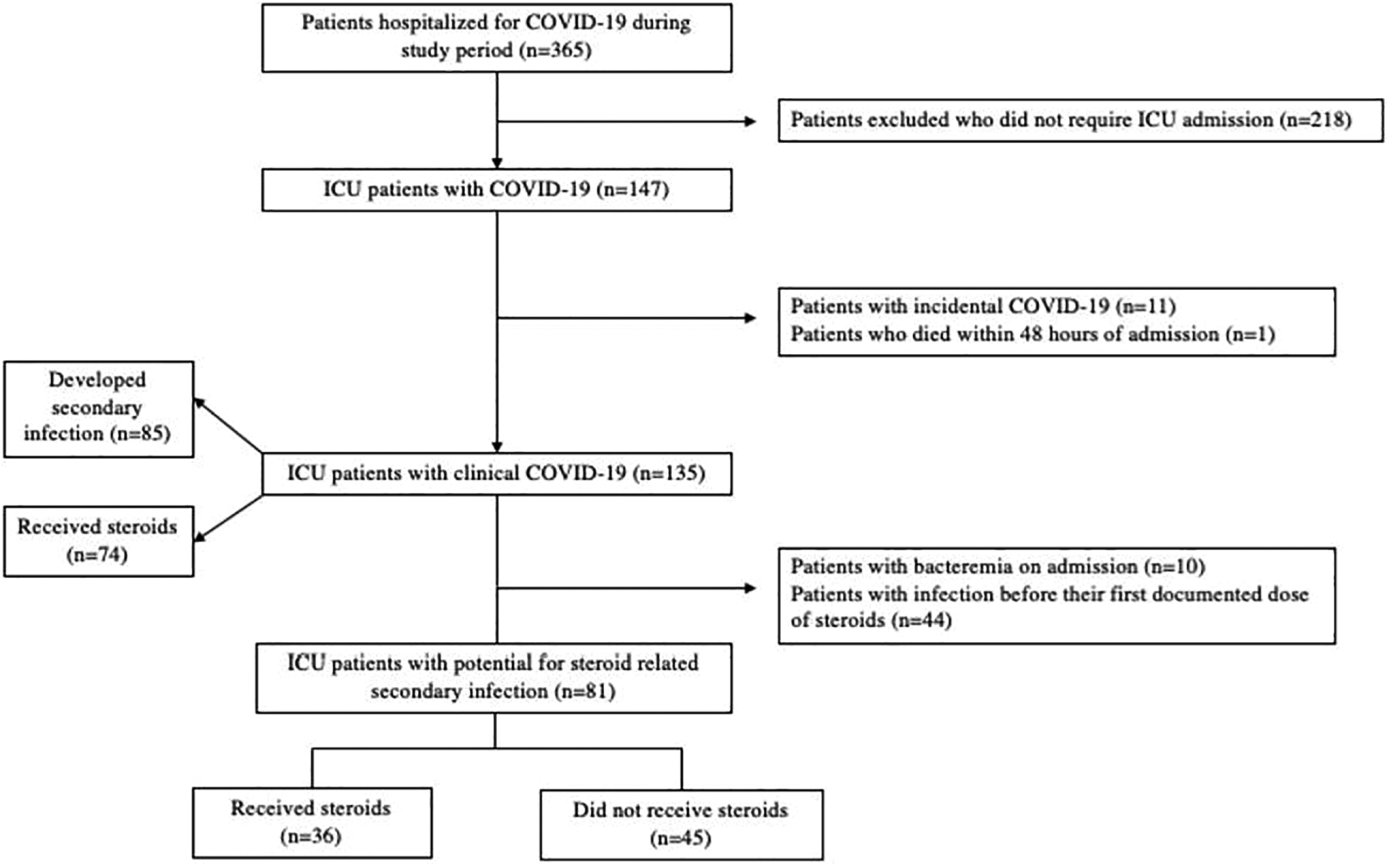
Study inclusion. Study consort diagram.

From March through June 2020, 365 patients were hospitalized at UMMC for COVID-19, of which 147 were admitted to the ICU. Twelve patients were excluded from the analysis due to death within 48 hours (n = 1) or incidental COVID-19 infection (n = 11) (Figure 1). The mean age of the patients in the cohort was 52.3 years, 29.6% were female, 33.3% were Black or African American, 29.6% were White, 1.5% were Asian, 0.7% were Native Hawaiian or Other Pacific Islander, and 26% were designated as other. Fifty (37%) patients were Hispanic or Latino.

### Impact of Corticosteroids on Secondary Infection

The proportion of patients with secondary infection during their hospitalization was 63% (n = 85). The average time from hospital admission, defined as admission to the initial presenting hospital, to positive culture data was 16.7 days, 11.2 days, and 20.8 days for blood, lower respiratory tract, and urine cultures, respectively (Supplement 1). The primary steroid utilized was methylprednisolone (67%) dosed via the protocol described by Meduri,^15,16^ followed by hydrocortisone (18.6%), dexamethasone (8.5%), betamethasone (5.7%), and prednisone (2.9%). Due to the timing of the data collection, no patients received dexamethasone dosing per the RECOVERY trial. Among all individuals who received corticosteroids, the average prednisone equivalent dose of steroids administered was 1360 mg with a mean 12.9 days of >20 mg prednisone equivalent doses.

**Figure 2.**
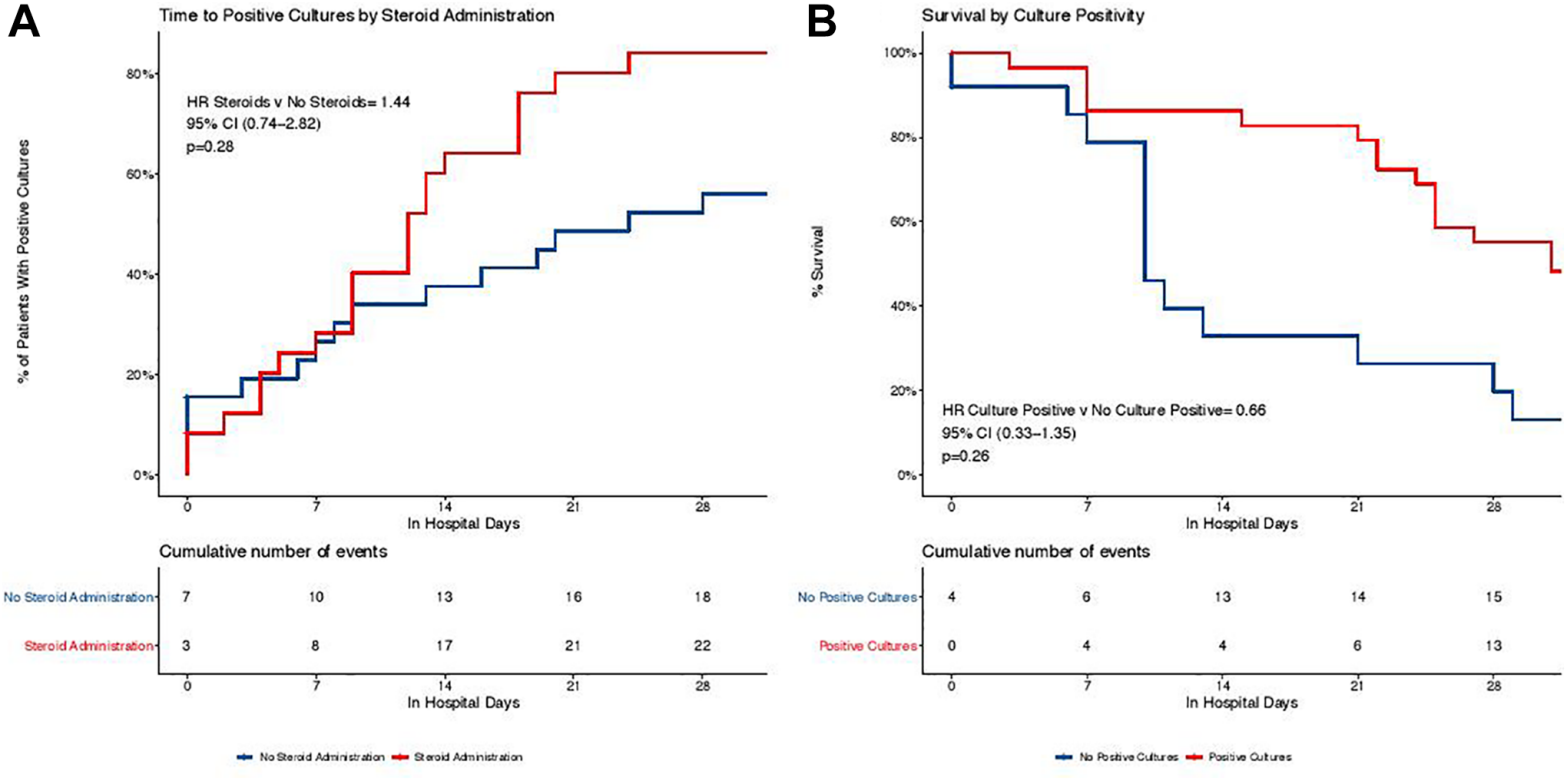
Kaplan-Meier estimates of secondary infection. A, Unadjusted Kaplan-Meier curves examining time to positive culture by corticosteroid administration. A fine-gray model was used to calculate the adjusted HR, which is adjusted for age. B, Unadjusted Kaplan-Meier curves examining time to mortality by the presence of secondary infection. The adjusted HR is adjusted for age.

In the subsequent time to secondary infection analysis, 54 of the 135 critically ill COVID-19 patients were excluded as they received corticosteroids within 48 hours of or after developing a secondary infection. The mean age of the patients included in the time to secondary infection analysis was 53.5 years, 35.8% were female, 2.5% Asian American, 37% African American, 37% White, 18.5% other, and 35.8% Hispanic or Latino (Table 1). Patients who received corticosteroids before developing infection received an average of 1210 mg of prednisone equivalent dose and received more than 20 mg equivalents of prednisone for an average of 11.9 days. The odds that a patient with any positive culture data was exposed to steroids was 1.96 (CI 0.81-4.79, *P* = 0.14). In the time to event analysis, there was no significant increase in the likelihood of secondary infection in patients who received corticosteroids (HR 1.45, CI 0.75-2.82, *P* = 0.28) when adjusting for age (Figure 2A). Similarly, there was no significant increase in the likelihood of bloodstream infection (HR 2.54, CI 0.85-7.58, *P* = 0.09), respiratory infection (HR 1.53, CI 0.65-0.72, *P* = 0.27), or urinary infection (HR 1.06, CI 0.29-3.90, *P* = 0.94).

**Table 1.** Demographics of Patients Who Received Corticosteroids Prior to Secondary Infection.

	All (N = 81)	Steroids (N = 36)	No steroids (N = 45)	*P*-value
Age (years)				0.002
Mean (SD)	53.5 (18.4)	46.4 (15.8)	59.1 (18.5)	
Median [Q1, Q3]	53.0 [41.0, 61.0]	44.5 [35.8, 60.5]	61.0 [48.0, 73.0]	
Female sex, N (%)	29 (35.8)	10 (27.8)	19 (42.2)	0.27
Race, N (%)				0.08
Asian American	2 (2.5)	1 (2.8)	1 (2.2)	
Black or African American	30 (37.0)	10 (27.8)	20 (44.4)	
White	30 (37.0)	12 (33.3)	18 (40.0)	
Other	15 (18.5)	11 (30.6)	4 (8.9)	
Hispanic or Latino, N (%)	29 (35.8)	17 (47.2)	12 (26.7)	0.13

### Impact of Secondary Infection on Mortality

The baseline demographics of all patients who developed a secondary infection are depicted in Table 2. The presence of secondary infection was not associated with the likelihood of 28-day mortality in an age-adjusted Cox proportional hazard estimate (HR 0.66, CI 0.33-1.35, *P* = 0.26) (Figure 2B). Similarly, the likelihood of mortality was not associated with the presence of bloodstream infection (HR 0.59, CI 0.31-1.13, *P* = 0.11), respiratory infection (HR 0.59, CI 0.30-1.19, *P* = 0.14), or urinary infection (HR 0.69, CI 0.30-1.58, *P* = 0.38), when adjusting for age.

**Table 2.** Demographics of Patients Who Developed Secondary Infection.

	All (N = 135)	Co-infection (N = 85)	No co-infection (N = 50)	*P*-value
Age (years)				0.53
Mean (SD)	52.3 (17.4)	51.9 (14.3)	53.3 (22.4)	
Median [Q1, Q3]	52.0 [40.0, 60.0]	51.0 [41.0, 62.0]	57.0 [35.0, 72.0]	
Female sex, N (%)	40 (29.6)	23 (27.1)	17 (34.0)	0.22
Race, N (%)				0.44
Asian American	2 (1.5)	1 (1.2)	1 (2.0)	
Black or African American	45 (33.3)	26 (30.6)	19 (38.0)	
White	40 (29.6)	26 (30.6)	14 (28.0)	
Native Hawaiian/Pacific Islander	1 (0.7)	1 (1.2)	0 (0)	
Other	26 (19.3)	21 (24.7)	5 (10.0)	
Multiple races	2 (1.5)	1 (1.2)	1 (2.0)	
Hispanic or Latino, N (%)	50 (37.0)	38 (44.7)	12 (24.0)	0.14

### Impact of Corticosteroids on Mortality

**Figure 3.**
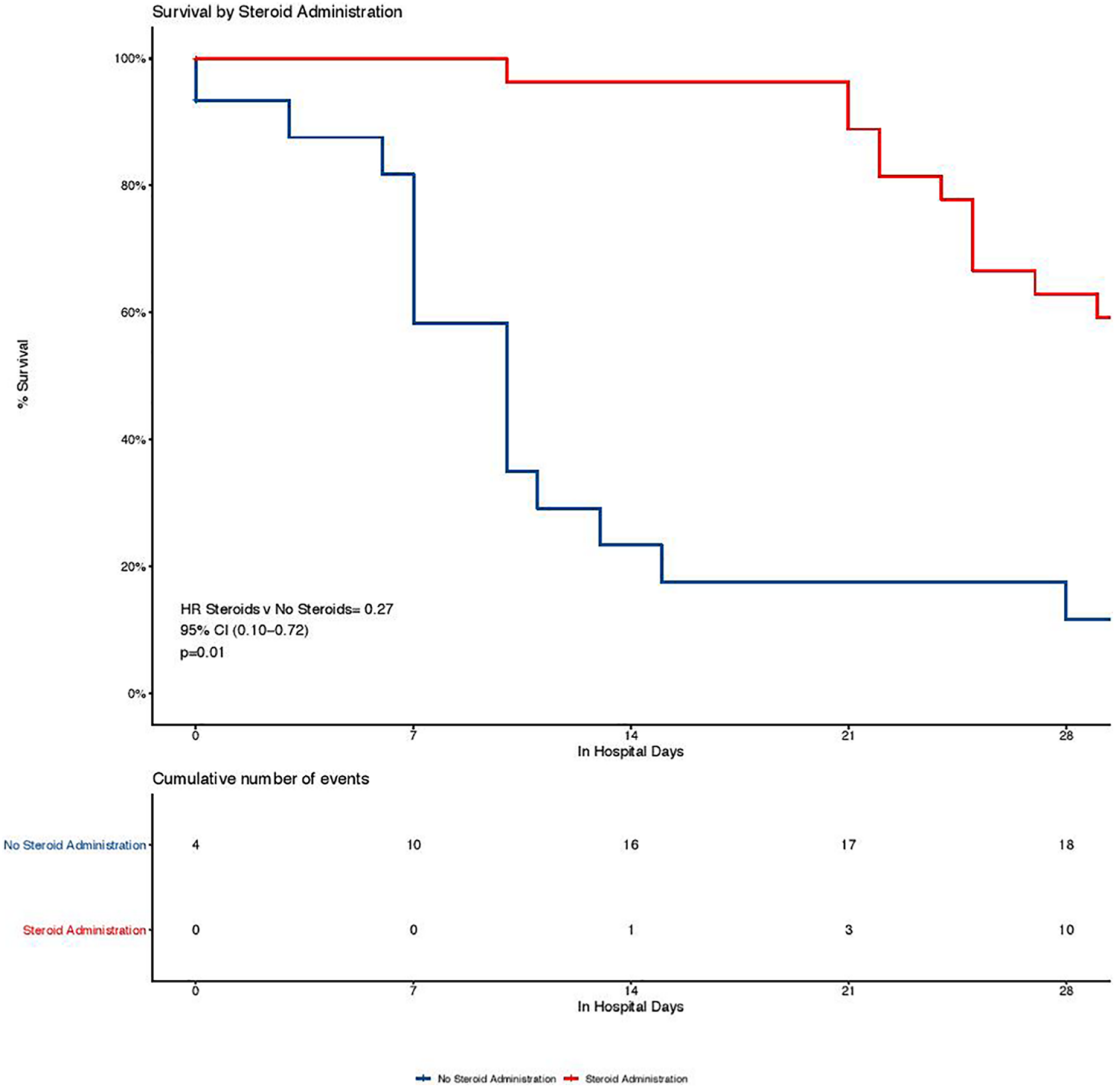
Survival by corticosteroid administration. Unadjusted Kaplan-Meier curve examining time to mortality by corticosteroid administration. A Cox-Proportional Hazard Model was used to calculate the adjusted HR, which is adjusted for age.

Seventy-four patients (55%) received corticosteroids during their hospitalization (Table 3). An unadjusted Cox proportional hazard estimate of 28-day mortality indicates that there is a 68% reduction in the likelihood of mortality during the 28-day period in patients treated with corticosteroids (HR 0.32, 0.17-0.58 *P* < 0.001) (Figure 3). This finding remains significant in an age-adjusted Cox proportional hazard estimate (HR 0.27, CI 0.10-0.72, *P* = 0.01).

**Table 3.** Demographics of All Patients Who Received Corticosteroids.

	All (N = 135)	Steroids (N = 74)	No steroids (N = 61)	*P*-value
Age (years)				0.01
Mean (SD)	52.3 (17.4)	49.2 (14.7)	54.2 (20.9)	
Median [Q1, Q3]	52.0 [40.0, 60.0]	48.5 [39.3, 60.0]	59.0 [35.0, 72.0]	
Female sex, N (%)	40 (29.6)	16 (21.6)	24 (39.3)	0.01
Race, N (%)				0.12
Asian American	2 (1.5)	1 (1.4)	1 (1.6)	
Black or African American	45 (33.3)	21 (28.4)	24 (39.3)	
White	40 (29.6)	22 (29.7)	18 (29.5)	
Native Hawaiian/Pacific Islander	1 (0.7)	1 (1.4)	0 (0)	
Other	26 (19.3)	21 (28.4)	5 (8.2)	
Multiple races	2 (1.5)	1 (1.4)	1 (1.6)	
Hispanic or Latino, N (%)	50 (37.0)	36 (48.6)	14 (23.0)	0.05

## Discussion

This study shows that critically ill SARS-CoV-2 patients have a high proportion of secondary infection (63%). Previous studies have estimated that the proportion of secondary infection varies from 5% to 40%, with critically ill patients falling toward the higher end of that range.^2,5–7,9,12,20–22,28–30^ We hypothesize that the increased proportion of secondary infection in our population is related to the higher level of critical illness. All patients included in this study required ICU level of care. As a whole, 83% of patients were mechanically ventilated, 7.4% required non-invasive positive pressure ventilation, 31.9% required high flow nasal cannula. Neuromuscular blockade was utilized in 43% of patients and 49.6% of patients were placed in the prone position. Twenty-four (17.8%) patients required extracorporeal membrane oxygenation (ECMO). Patients had prolonged ICU and hospital lengths of stay, averaging 22.4 and 26.8 days, respectively. Therefore, this population reflects an exceedingly high acuity population, which allows us to establish the rate of secondary infection among the sickest of the critically ill patients.

Secondary infection research is also subject to an important epidemiological principle known as competing risks. This concept is imperative in COVID-19 where the rate of mortality is high (>35% in ICU patients in our study) and frequently occurred relatively early in the disease process, with over 30% of deaths occurring in the first two weeks of the hospitalization. Without accounting for competing risks, the previously published studies assessing risk of secondary infection are likely inaccurate.^31^ Our work utilized the Fine-Grey competing risk model to account for this epidemiological principle and, thus, allowed for more accurate exploration of the relationship between corticosteroids and secondary infection. It also provided an improved estimate of time to secondary infection.

Prior to COVID-19, the safety and effectiveness of corticosteroids for ARDS were debated.^14–16,32,33^ The RECOVERY trial, while practice changing in terms of bringing corticosteroids use to the forefront of COVID-19 therapeutics, did not report the incidence of secondary infections.^13^ Our work specifically evaluates whether corticosteroid use is associated with the rate of secondary infection in critically ill COVID-19 patients and found no increase in the likelihood of bloodstream, respiratory tract, or urinary infections in patients treated with corticosteroids. Our findings parallel two recently published randomized clinical trials, which administered corticosteroids in COVID-19 and explored secondary infection as a secondary outcome.^6,7^ While these trials were terminated early due to the publication of the RECOVERY trial, they found no increase in secondary infection in patients treated with corticosteroids.^6,7^ Furthermore, despite the high proportion of secondary infection in our study cohort, secondary infection was not significantly associated with the rate of 28-day mortality. These results provide reassurance to clinicians apprehensive of the infection risk of corticosteroid use in COVID-19.

Our data echoes the findings of the recently published RECOVERY trial and reinforces not just the safety but the efficacy of corticosteroids in the most critically ill patients hospitalized with COVID-19. The RECOVERY trial reported that mechanically ventilated patients with COVID-19 are 46% less likely to die if treated with dexamethasone when adjusting for age.^13^ In a Cox proportional model adjusted for age, we demonstrate a 73% reduction in 28-day case-fatality rate with steroid administration among our cohort. When additionally controlling for mechanical ventilation, our effect remains highly significant, demonstrating a 74% reduction in case fatality. Patients included in this analysis primarily received methylprednisolone followed by hydrocortisone, suggesting this mortality benefit may be a class effect rather than the effect of a particular corticosteroid. The survival benefit of corticosteroids on 28-day case-fatality in the most critically ill patients, amid the absence of evidence to suggest that corticosteroids are associated with secondary infection or that secondary infection worsens patient survival, tips the scales heavily in favor of the use of corticosteroids for the critically ill patient with COVID-19.

Our work was able to further support corticosteroids as a therapeutic option for COVID-19 but was unable to elucidate the exact etiology of the high proportion of secondary infections in critically ill patients with COVID-19. It is uncertain what role the SARS-CoV-2 virus itself has played immunologically in the propensity for secondary infections in COVID-19. The rate of secondary infection as well as the dramatic and reproducible mortality benefit of corticosteroids in COVID-19 points to a distinct phenotype of ARDS, which benefits from the use of anti-inflammatory therapy.^13,18,34^ Besides immunological properties of the virus itself, COVID-19 related logistics are likely contributory to the high proportion of secondary infection. Patients are often cared for in negative pressure units rather than negative pressure rooms and based on available resources, multiple ICU patients may be cohorted in a single room. This has the potential to propagate infection transmitted by providers and equipment between patients. There are no clear guidelines on how to maintain traditional contact precautions in a negative pressure unit while donned in full personal protective equipment. The exceedingly high proportion of secondary infection, therefore, may be related to a yet unidentified property of the SARS CoV-2 virus compounded by evolving infection prevention strategies.

The generalizability of this study is limited in that it is a single-center retrospective cohort study conducted in an urban hospital in the United States. Corticosteroid administration strategies and infection rates are subject to institution-specific practices. For example, the UMMC was forced to cohort patients at the height of the COVID-19 pandemic in negative pressure units. The role that this played in the rate of secondary infection is not yet defined. Regarding the analysis of the association between corticosteroids with the outcomes of secondary infection and mortality, no patients enrolled in the study died within the first 48 hours of inclusion and all patients with the outcome of secondary infection received corticosteroids at least 48 hours prior to their first positive culture. Thus, each patient had ample opportunity to receive corticosteroids. In fact, approximately 30% of patients enrolled received corticosteroids within the first 24 hours of presentation to the hospital (Supplement 2). This significantly diminishes concern for immortal time bias in our analysis.

The results of this study are strengthened by the high level of acuity of the population, the relatively large number of patients included in the analysis, as well as the rigorous definition of secondary infection. Furthermore, the statistical analysis appropriately accounted for competing risks, resulting in a more accurate estimation of the time to secondary infection. Our findings contribute to the growing body of research exploring corticosteroid treatment in COVID-19. Further studies should focus on longer term outcomes of both morbidity and mortality in critically ill patients receiving corticosteroids, whether the addition of COVID-19 directed therapies (i.e., tocilizumab) contributes to secondary infection, and the risk of corticosteroid use in regions where fungal infection may be more prevalent.

## Conclusion

The development of secondary infections is a commonly feared corticosteroid-related complication. The results of this study should conciliate those fears. Furthermore, the presence of a secondary infection does not increase the COVID-19 case fatality. The use of corticosteroids, a lifesaving therapy for many critically ill patients with COVID-19, should be accompanied by heightened awareness but not trepidation regarding the risk of secondary infection.

## Supplemental Material

Supplemental Material, sj-pdf-1-jic-10.1177_08850666211032175 - The Impact of Corticosteroids on Secondary Infection and Mortality in Critically Ill COVID-19 Patients Click here for additional data file. Supplemental Material, sj-pdf-1-jic-10.1177_08850666211032175 for The Impact of Corticosteroids on Secondary Infection and Mortality in Critically Ill COVID-19 Patients by Lindsay A. Ritter, Noel Britton, Emily L. Heil, William A. Teeter, Sarah B. Murthi, Jonathan H. Chow, Emily Ricotta, Daniel S. Chertow, Alison Grazioli and Andrea R. Levine in Journal of Intensive Care Medicine

Supplemental Material, sj-pdf-2-jic-10.1177_08850666211032175 - The Impact of Corticosteroids on Secondary Infection and Mortality in Critically Ill COVID-19 Patients Click here for additional data file. Supplemental Material, sj-pdf-2-jic-10.1177_08850666211032175 for The Impact of Corticosteroids on Secondary Infection and Mortality in Critically Ill COVID-19 Patients by Lindsay A. Ritter, Noel Britton, Emily L. Heil, William A. Teeter, Sarah B. Murthi, Jonathan H. Chow, Emily Ricotta, Daniel S. Chertow, Alison Grazioli and Andrea R. Levine in Journal of Intensive Care Medicine

## References

[1] Yang X Yu Y Xu J, et al. Clinical course and outcomes of critically ill patients with SARS-CoV-2 pneumonia in Wuhan, China: a single-centered, retrospective, observational study. Lancet Respir Med. 2020;8(5):475–481. 32105632 10.1016/S2213-2600(20)30079-5 PMC7102538

[2] Wang D Hu B Hu C, et al. Clinical characteristics of 138 hospitalized patients with 2019 novel coronavirus-infected pneumonia in Wuhan, China. JAMA. 2020;323(11):1061–1069. 32031570 10.1001/jama.2020.1585 PMC7042881

[3] Chertow DS Memoli MJ. Bacterial coinfection in influenza: a grand rounds review. JAMA. 2013;309(3):275–282. 23321766 10.1001/jama.2012.194139

[4] Zhou F Yu T Du R, et al. Clinical course and risk factors for mortality of adult inpatients 333 with COVID-19 in Wuhan, China: a retrospective cohort study. Lancet. 2020;395(10229):1054–1062. 32171076 10.1016/S0140-6736(20)30566-3 PMC7270627

[5] Lansbury L Lim B Baskaran V, et al. Co-infections in people with COVID-19: a systematic review and meta-analysis. J Infect. 2020;81(2):266–275. 32473235 10.1016/j.jinf.2020.05.046 PMC7255350

[6] Tomazini BM Maia IS Cavalcanti AB, et al. Effect of dexamethasone on days alive and ventilator-free in patients with moderate or severe acute respiratory distress syndrome and COVID-19: the CoDEX randomized clinical trial. JAMA. 2020;324(13):1307–1316. 32876695 10.1001/jama.2020.17021 PMC7489411

[7] Dequin PF Heming N Meziani F, et al. Effect of hydrocortisone on 21-day mortality or respiratory support among critically ill patients with COVID-19: a randomized clinical trial. JAMA. 2020;324(13):1298–1306. 32876689 10.1001/jama.2020.16761 PMC7489432

[8] Rouzé A Martin-Loeches I Povoa P, et al. Relationship between SARS-CoV-2 infection and the incidence of ventilator-associated lower respiratory tract infections: a European multicenter cohort study. Intensive Care Med. 2021;47(2):188–198. 33388794 10.1007/s00134-020-06323-9 PMC7778569

[9] Rawson TM Moore LSP Zhu N, et al. Bacterial and fungal co-infection in individuals with coronavirus: a rapid review to support COVID-19 antimicrobial prescribing. Clin Infect Dis. 2020;71(9):2459–2468. 32358954 10.1093/cid/ciaa530 PMC7197596

[10] Mehta P McAuley DF Brown M, et al. COVID-19: consider cytokine storm syndromes and immunosuppression. Lancet. 2020;395(10229):1033–1034. 32192578 10.1016/S0140-6736(20)30628-0 PMC7270045

[11] Ritchie AI Singanayagam A. Immunosuppression for hyperinflammation in COVID-19: a double-edged sword? Lancet. 2020;395(10230):1111. 10.1016/S0140-6736(20)30691-7 PMC7138169 32220278

[12] Lin D Liu L Zhang M, et al. Co-infections of SARS-CoV-2 with multiple common respiratory pathogens in infected patients. Sci China Life Sci. 2020;63(4):606–609. 32170625 10.1007/s11427-020-1668-5 PMC7089461

[13] RECOVERY Collaborative Group; Horby P Lim WS, et al. Dexamethasone in hospitalized patients with COVID-19. N Engl J Med. 2020;384(8):693–704. 32678530 10.1056/NEJMoa2021436 PMC7383595

[14] Villar J Ferrando C Martínez D, et al. Dexamethasone treatment for the acute respiratory distress syndrome: a multicentre, randomised controlled trial. Lancet Respir Med. 2020;359(8):267–276. 10.1016/S2213-2600(19)30417-5 32043986

[15] Meduri GU Headley AS Golden E, et al. Effect of prolonged methylprednisolone therapy in unresolving acute respiratory distress syndrome: a randomized controlled trial. JAMA. 1998;280(2):159–165. 9669790 10.1001/jama.280.2.159

[16] Meduri GU Golden E Freire AX, et al. Methylprednisolone infusion in early severe ARDS: results of a randomized controlled trial. Chest. 2007;131(4):954–963. 17426195 10.1378/chest.06-2100

[17] Belvitch P Dudek SM. Corticosteroids and acute respiratory distress syndrome: the debate continues. Crit Care Med. 2013;41(7):1813–1814. 23774346 10.1097/CCM.0b013e3182963cfb PMC3703846

[18] WHO Rapid Evidence Appraisal for COVID-19 Therapies (REACT) Working Group; Sterne JAC Murthy S, et al. Association between administration of systemic corticosteroids and mortality among critically ill patients with COVID-19: a meta-analysis. JAMA. 2020;324(13):1330–1341. 32876694 10.1001/jama.2020.17023 PMC7489434

[19] Angus DC Derde L Al-Beidh F, et al. Effect of hydrocortisone on mortality and organ support in patients with severe COVID-19: the remap-cap COVID-19 corticosteroid domain randomized clinical trial. JAMA. 2020;324(13):1317–1329. 32876697 10.1001/jama.2020.17022 PMC7489418

[20] Hughes S Troise O Donaldson H, et al. Bacterial and fungal coinfection among hospitalized patients with COVID-19: a retrospective cohort study in a UK secondary-care setting. Clin Microbiol Infect. 2020;26(10):1395–1399. 32603803 10.1016/j.cmi.2020.06.025 PMC7320692

[bibr21-08850666211032175] Adler H Ball R Fisher M Mortimer K Vardhan MS. Low rate of bacterial co-infection in patients with COVID-19. Lancet Microbe. 2020;1(2):e62. 32835331 10.1016/S2666-5247(20)30036-7 PMC7279742

[22] Zhu X Ge Y Wu T, et al. Co-infection with respiratory pathogens among COVID-2019 cases. Virus Res. 2020;285:198005. 32408156 10.1016/j.virusres.2020.198005 PMC7213959

[23] Brun-Buisson C Richard J-CM Mercat A, et al. Early corticosteroids in severe influenza A/H1N1 pneumonia and acute respiratory distress syndrome. Am J Respir Crit Care Med. 2011;183(9):1200–1206. 21471082 10.1164/rccm.201101-0135OC

[24] McHugh ML. The chi-square test of independence. Biochem Med (Zagreb). 2013;23(2):143–149. 23894860 10.11613/BM.2013.018 PMC3900058

[25] Kruskal WH. Historical notes on the Wilcoxon unpaired two-sample test. J Am Stat Assoc. 1957;52:356–360.

[26] Fine JP Gray RJ. A proportional hazards model for the subdistribution of a competing risk. J Am Stat Assoc. 1999;94(446):496–509.

[27] Enderlein G Cox DR Oakes D. Analysis of survival data. Chapman and Hall. London New York 1984, 201 S., £ 12,–. Biom J. 1987;29:114–114.

[28] Goyal P Choi JJ Pinheiro LC, et al. Clinical characteristics of COVID-19 in New York City. N Engl J Med. 2020;382(24):2372–2374. 32302078 10.1056/NEJMc2010419 PMC7182018

[bibr29-08850666211032175] Zangrillo A Landoni G Biondi-Zoccai G, et al. A meta-analysis of complications and mortality of extracorporeal membrane oxygenation. Crit Care Resusc. 2013;15(3):172–178. 23944202

[30] Zangrillo A Beretta L Scandroglio AM, et al. Characteristics, treatment, outcomes and cause of death of invasively ventilated patients with COVID-19 ARDS in Milan, Italy. Crit Care Resusc. 2020;22(3):200–211. 32900326 10.1016/S1441-2772(23)00387-3 PMC10692521

[31] Bassetti M Kollef MH Timsit JF. Bacterial and fungal superinfections in critically ill patients with COVID-19. Intensive Care Med. 2020;46(11):2071–2074. 32902729 10.1007/s00134-020-06219-8 PMC7479998

[32] Steinberg KP Hudson LD Goodman RB, et al. Efficacy and safety of corticosteroids for persistent acute respiratory distress syndrome. N Engl J Med. 2006;354(16):1671–1684. 16625008 10.1056/NEJMoa051693

[33] Prescott HC Rice TW. Corticosteroids in COVID-19 ARDS: evidence and hope during the pandemic. JAMA. 2020;324(13):1292–1295. 32876693 10.1001/jama.2020.16747

[34] Matthay MA Arabi YM Siegel ER, et al. Phenotypes and personalized medicine in the acute respiratory distress syndrome. Intensive Care Med. 2020;46(12):2136–2152. 33206201 10.1007/s00134-020-06296-9 PMC7673253

