# Supplementary material for: The Impact of Corticosteroids on Secondary Infection and Mortality in Critically Ill COVID-19 Patients
Source: J Intensive Care Med. 2021 Oct;36(10):1201–8. doi: 10.1177/08850666211032175 (PMC8442131; doi:10.1177/08850666211032175)
Supplement: Supplemental Material, sj-pdf-1-jic-10.1177_08850666211032175 - The Impact of Corticosteroids on Secondary Infection and Mortality in Critically Ill COVID-19 Patients [file sj-pdf-1-jic-10.1177_08850666211032175.pdf]

Supplement 1. Pathogen by culture site.

Culture Site	Pathogen	Number of Positive Cultures
**Blood**		N=63 (%)
	Coagulase negative staph	10 (15.87)
	Enterococcus faecalis	10 (15.87)
	Staphylococcus aureus	5 (7.93)
	Streptococcus pneumoniae	1 (1.59)
	Streptococcus anginosis	2 (3.17)
	Staphylococcus schleiferi	1 (1.59)
	Group A streptococcus pyogenes	1 (1.59)
	Streptococcus constellatus	1 (1.59)
	Streptococcus mitus	1 (1.59)
	Bacillus cereus	2 (3.17)
	Klebsiella species	5 (7.93)
	Pseudomonas aeruginosa	4 (6.35)
	Escherichia coli	7 (11.11)
	Serratia marcescens	1 (1.59)
	Acinetobacter baumannii	2 (3.17)
	Ralstonia picketti	1 (1.59)
	Candida albicans	7 (11.11)
	Candida glabrata	1 (1.59)
	Candida parapsilosis	1 (1.59)
**Sputum**		N=111 (%)
	Staphylococcus aureus	25 (22.52)
	Alpha hemolytic strep	4 (3.60)
	Group A streptococcus pyogenes	1 (1)
	Streptococcus pneumonia	1 (1)
	Escherichia coli	16 (14.41)
	Klebsiella species	27 (24.32)
	Pseudomonas aeruginosa	17 (15.31)
	Proteus mirabilis	3 (2.70)
	Morganella morgani	1 (1)
	Serratia marcescens	7 (6.30)
	Enterobacter cloacea	1 (1)
	Acinetobacter bumannii	6 (5.40)
	Stenotrophomonas maltophia	2 (1.80)
**Urine**		N=16 (%)
	Escherichia coli	5 (31.25)
	Klebsiella species	6 (37.5)
	Pseudomonas aeruginosa	2 (12.5)
	Proteus mirabilis	1 (6.25)
	Serratia marcescens	1 (6.25)
	Enterococcus faecalis	1 (6.25)

Supplement 1. Pathogens by culture site. Coagulase negative staph species was included only when this was present in multiple blood culture bottles taken simultaneously with treatment suggested by the infectious disease specialists.
