# Supplementary material for: The Impact of Corticosteroids on Secondary Infection and Mortality in Critically Ill COVID-19 Patients
Source: J Intensive Care Med. 2021 Oct;36(10):1201–8. doi: 10.1177/08850666211032175 (PMC8442131; doi:10.1177/08850666211032175)
Supplement: Supplemental Material, sj-pdf-2-jic-10.1177_08850666211032175 - The Impact of Corticosteroids on Secondary Infection and Mortality in Critically Ill COVID-19 Patients [file sj-pdf-2-jic-10.1177_08850666211032175.pdf]

Supplement 2. Time to corticosteroid administration.

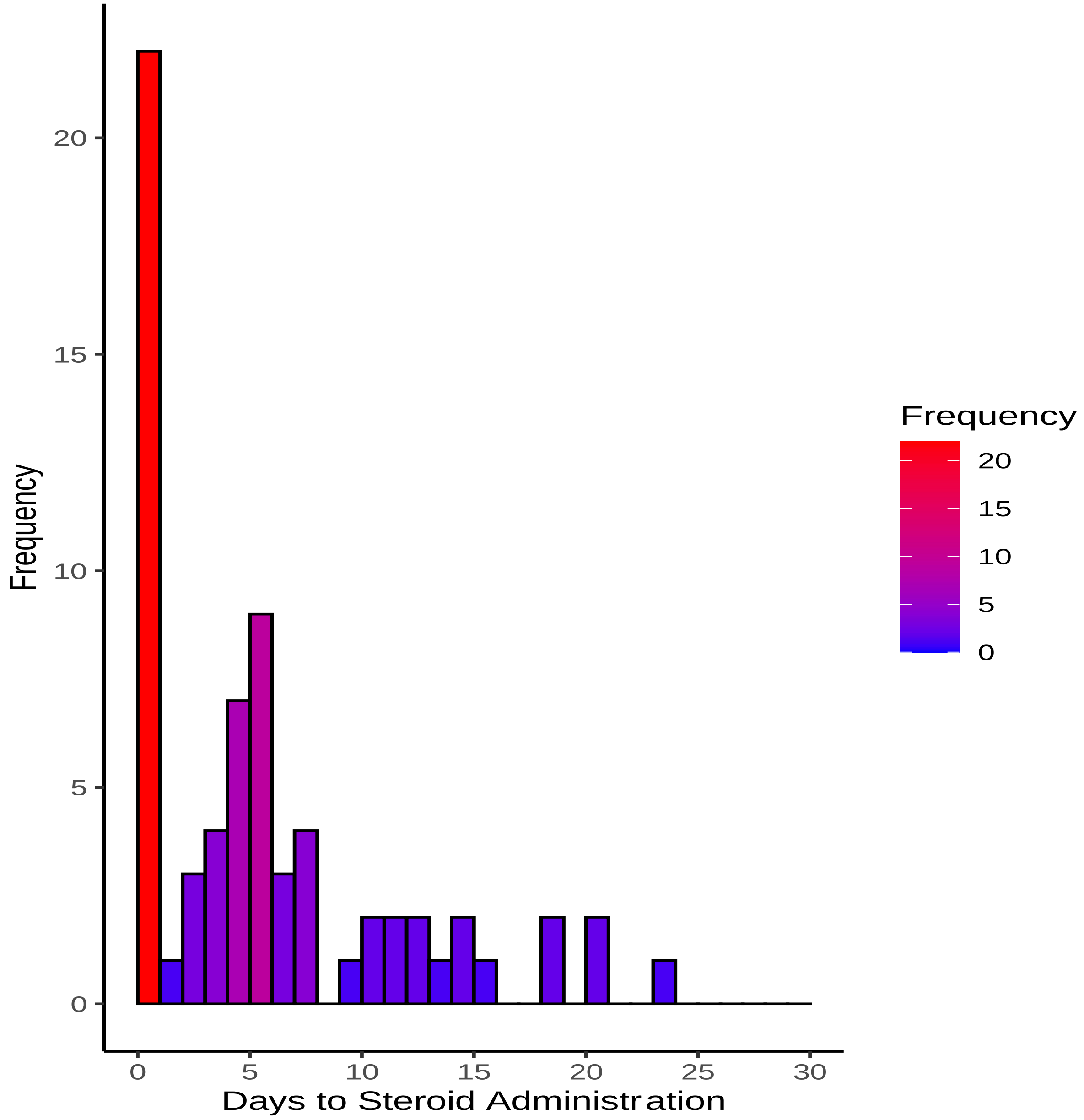

Supplement 2. Histogram depicting time to first corticosteroid dose in days.
